# Enhanced Dissolution and Oral Bioavailability of Piroxicam Formulations: Modulating Effect of Phospholipids

**DOI:** 10.3390/pharmaceutics2040339

**Published:** 2010-10-27

**Authors:** Sabiruddin Mirza, Inna Miroshnyk, Muhammad J. Habib, James F. Brausch, Muhammad D. Hussain

**Affiliations:** 1 Division of Pharmaceutical Technology, Faculty of Pharmacy, University of Helsinki, P.O. Box 56, FIN- 00014, University of Helsinki, Finland; 2 School of Pharmacy, Howard University, 2300 Fourth Street, NW, Washington, DC 20059, USA; 3 Irma Lerma Rangel College of Pharmacy, Texas A&M Health Sciences Center, MSC 131, 1010 West Avenue B, Kingsville, TX 78363, USA

**Keywords:** poorly water soluble drugs, phospholipids, solid dispersions, dissolution, bioavailability

## Abstract

Several biologically relevant phospholipids were assessed as potential carriers/additives for rapidly dissolving solid formulations of piroxicam (Biopharmaceutics Classification System Class II drug). On the basis of *in vitro* dissolution studies, dimyristoylphosphatidylglycerol (DMPG) was ranked as the first potent dissolution rate enhancer for the model drug. Subsequently, the solid dispersions of varying piroxicam/DMPG ratios were prepared and further investigated. Within the concentration range studied (6.4-16.7 wt %), the dissolution rate of piroxicam from the solid dispersions appeared to increase as a function of the carrier weight fraction, whereas the cumulative drug concentration was not significantly affected by piroxicam/DMPG ratio, presumably due to a unique phase behavior of the aqueous dispersions of this carrier phospholipid. Solid state analysis of DMPG-based formulations reveled that they are two-component systems, with a less thermodynamically stable form of piroxicam (Form II) being dispersed within the carrier. Finally, oral bioavailability of piroxicam from the DMPG-based formulations in rats was found to be superior to that of the control, as indicated by the bioavailability parameters, *c*_max _ and especially *T*_max_ (53 μg/mL within 2 h *vs.* 39 μg/mL within 5.5 h, respectively). Hence, DMPG was regarded as the most promising carrier phospholipid for enhancing oral bioavailability of piroxicam and potentially other Class II drugs.

## 1. Introduction

Sufficient aqueous solubility is a prerequisite for effective oral delivery of any therapeutic agent. However, low soluble and highly permeable drug molecules are gradually becoming prevailing in the development pipelines of pharmaceutical companies. Such drug molecules fall into Biopharmaceutics Classification System (BCS) Class II [1], for which the dissolution is usually the rate-limiting step for gastrointestinal absorption. To enhance dissolution rate and thus oral absorption of the Class II drugs numerous formulation strategies have been developed. These encompass salt formation [2], micro- and nanosizing [3], and solid dispersions [4,5,6], to name a few. The latter approach is recently regaining interest within the pharmaceutical industry, especially in the context of amorphous and nanoparticulate formulations [7,8,9,10].

Traditionally, freely water-soluble polymers such as high molecular weight polyethylene glycols (PEG) and polyvinylpyrrolidones (PVP) have been utilized as carriers for solid dispersion formulations [11,12]. However, the literature search has revealed that lipid-based systems demonstrated a higher success rate in enhancing the bioavailability of Class II drugs [1,5]. Phospholipids are the most promising group of carriers that has been probed for lipid-based formulations [13,14,15]. Phospholipids are amphiphilic compounds and, when equilibrated with excess water, spontaneously form bilayer structures (liposomes) having the capacity to entrap drug solutes. The drug partitioned in these bilayer structures of high surface area is transported to the stationary or diffusion layer yielding an increased rate of dissolution into aqueous environment [16]. Moreover, the use of lipids and lipid-like compounds as drug carriers is thought to promote oral absorption via intrinsic lipid pathways [5]. Finally, the severity of some adverse effects – for instance, the gastrointestinal tract ulceration and bleeding associated with non-steroidal anti-inflammatory drugs (NSAIDs) - can be reduced through lipid-based formulations [16,17].

Piroxicam is an NSAID that belongs to the BCS Class II drugs and thus its oral absorption is considered to be dissolution-rate limited [1,18]. In fact, several studies have reported a significant increase in the dissolution rate of piroxicam solid dispersions over the pure drug formulations [19,20,21]. In these systems, mainly PEG and PVP have been used as drug carriers and only the recent study [22] has exploited *in vitro* dissolution and permeability of phospholipid-based solid dispersions. Nevertheless, the *in vivo* performance of lipid-based formulations of piroxicam has not received much attention in the literature.

Hence, the present study was designed to compare the pharmacokinetic properties of piroxicam delivered as pure drug and phospholipid-based solid dispersions and thus evaluate the potential of these delivery systems for oral bioavailability enhancement of the model drug. Several biologically relevant phospholipids (dimyristoylphosphatidylglycerol, dimyristoylphosphatidylcholine, dipalmitoylphosphatidylcholine, and distearoylphosphatidylcholine) were initially selected as drug carriers for solid dispersion formulations and probed *in vitro*. The most potent phospholipid (dimyristoylphosphatidylglycerol) —in terms of dissolution rate enhancement—was then used as the carrier for formulation optimization and *in vivo* assessment. In addition, solid-state properties of the phospholipid-based formulations were studied to gain a better understanding of the relationship between the drug physical state and formulation performance.

## 2. Experimental Section

### 2.1. Materials

Piroxicam, dimyristoylphosphatidylglycerol (DMPG), dimyristoylphosphatidylcholine (DMPC), dipalmitoylphosphatidylcholine (DPPC), and distearoylphosphatidylcholine (DSPC) were purchased from Sigma Chemicals (St. Louis, MO) and were used as received. The HPLC grade solvents used throughout the study were obtained from Fisher Scientific.

### 2.2. Preparation of Phospholipid-Based Solid Dispersions and Physical Mixture

Solid dispersions of piroxicam and phospholipids were prepared by a solvent method. Specifically, piroxicam and required amount of a phospholipid (DMPC, DMPG, DPPC or DSPC) were subsequently dissolved in chloroform with gentle stirring. Chloroform was removed at room temperature under nitrogen and the solids were dried in a vacuum desiccator overnight. Solid dispersions were tested within 48 hours after preparation. Control piroxicam was prepared in the same manner with no phospholipid added. Physical mixtures were prepared by gently triturating appropriate quantities of piroxicam and a phospholipid.

### 2.3. Dissolution Studies

Prior to dissolution studies, the samples were passed through an 80-mesh sieve. The dissolution studies were performed using VK7000 dissolution test apparatus with an external temperature control unit and VK8000 automatic sample collector (VanKel Industries, Inc. Edison, NJ). An USP standard paddle continuously stirred the dissolution medium (900 mL of distilled deionized water maintained at 37 ºC) at 100 rpm. The powders (containing 20 mg of piroxicam) were sprinkled on the surface of the stirred dissolution medium at the beginning of the study (time zero). Solid dispersion powders dispersed quickly and distributed homogeneously with the stirring. At designated time intervals, samples were taken by the automatic sample collector and the concentration of piroxicam dissolved was determined using a UV spectrophotometer at wavelength 360 nm (Spectronic 1201, Milton Roy), where no interference with the carrier phospholipids was observed. The initial dissolution rate was calculated over the first 5 min of the test.

### 2.4. Differential Scanning Calorimetry (DSC)

Differential scanning calorimetry curves were obtained with a Differential Scanning Calorimeter 2010 (TA Instruments, Inc. New Castle, DE). About 2-4 mg of sample was loaded into an aluminum sample pan for each run. The thermal cycle was performed using a 10 ºC/min heating rate under an argon atmosphere from 25 to 300 ºC. The temperature and enthalpy of the signal were calibrated using the melting transition of indium (*T*_m_ = 156.6 ºC).

### 2.5. Powder X-ray Diffraction (PXRD)

Powder X-ray diffraction studies were done with a XDS2000 automated powder diffractometer (SCINTAG, CA). About 50 mg of each sample was run as a smear mount on a glass slide. X-ray diffraction patterns were obtained by using Cu-Kα radiation (λ = 1.54060 angstroms), 40 kV, 30 mA, 0.03 degrees/step and 1 degree 2-theta/min.

### 2.6. Bioavailability Studies

The oral absorption of piroxicam was determined in male Sprague-Dawley rats (weight 270-340 g). The external jugular vein of rats was cannulated. Animals were housed in individual cages, and given free access to food and water. One day after surgery, rats (n = 3) were given 20 mg/kg equivalent of piroxicam by oral administration. The vehicle was 1.0% hydroxypropylmethyl cellulose. Blood samples were drawn over 48 hours through the jugular vein and collected in Microtainer brand tube with lithium heparin. Samples were centrifuged immediately and the supernatant plasma was separated and stored at -70 ºC until analysis. Pharmacokinetic parameters were calculated using the computer program LAGRAN [23]. Differences in the pharmacokinetic parameters obtained between two groups were statistically evaluated using Student's unpaired t-test (p = 0.05).

### 2.7. HPLC Analysis

Plasma piroxicam concentrations were determined by a modified HPLC method [24]. Calibration samples were prepared by making the appropriate dilution of stock solution of piroxicam (1 mg/mL in 0.04 M phosphate buffer pH 8.0) in drug free rat plasma. Samples of plasma (0.1 mL) were placed in 20 mm × 150 mm glass culture tubes with screw caps; 50 μL of internal standard (Naproxen, 0.1 mg/mL in 0.04 M phosphate buffer pH 8.0), 0.2 mL of 1.0 M phosphate buffer (pH 2.0) and 5 mL of methylene chloride were added. The tubes were capped and shaken for 10 minutes and then centrifuged at 2000 ×g for 10 minutes. The aqueous layer was aspirated and the organic layer was dried under nitrogen. Samples were reconstituted with 0.1 mL mobile phase. Chromatographic separation was done on a Waters μBondapak (C18, 3.9 × 300 mm) column with a C18 precolumn insert. The mobile phase was 45% methanol in phosphate buffer (pH 8.0) and the flow rate was 1.0 mL/min. The UV detector (Waters 486) was set at 330 nm. An aliquot of 50 μL of the reconstituted solution was injected onto the HPLC column. The sensitivity of the method was about 50 g/mL.

## 3. Results and Discussion

### 3.1. Effect of the Carrier Phospholipid on Dissolution Rate of Piroxicam

**Figure 1:**
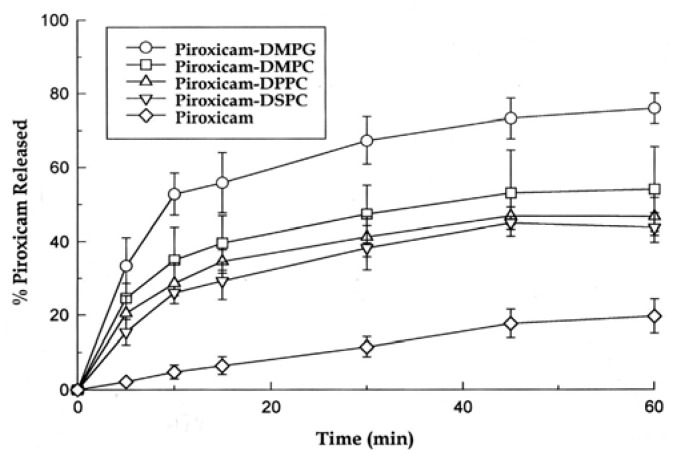
Dissolution profiles of the 10:1 (drug-to-carrier ratio) solid dispersions of piroxicam with various phospholipids in comparison with control piroxicam at 37 ºC in distilled water. Each data point refers to mean ± SD (n = 3).

In order to identify the most potent dissolution rate enhancer for the model drug, the 10:1 (drug-to-carrier ratio, w/w) binary solid dispersions of piroxicam with varying carrier phospholipids such as DMPC, DMPG, DPPC and DSPC were initially prepared and subsequently evaluated in terms of their dissolution performance. The data presented in Figure 1 clearly show that regardless of the carrier, the dissolution rate of the drug from the solid dispersions was significantly (up to a 17-fold) higher when compared to that of control piroxicam. Further, an analysis of the initial dissolution rates and limiting concentrations after 60 min yielded by the different solid dispersions (Table 1) was performed. This analysis made it possible to establish a rank order—DSPC< DPPC< DMPC<DMPG—of the carrier phospholipids with respect to their enhancing effect on the dissolution rate of piroxicam.

It is worth noting that the established rank order correlated well with the main phase transition temperature (*T*c) of the carrier phospholipids. More specifically, DPPC and DSPC as phospholipids with *T*c higher than 37 ºC [25] (*i.e.* the temperature of the dissolution medium) caused the minimal effect on the dissolution rate of piroxicam. Obviously, this can be attributed to the fact that under the present experimental conditions these phospholipids remained predominantly in the solid crystalline state. In contrast, DMPC and DMPG, the phospholipids with *T*c lower than 37 ºC [26], did undergo the phase transition at the experimental temperature and thus demonstrated the utmost enhancing effect on the dissolution kinetics of the model drug.

Hence, DMPG appeared to be the most promising carrier phospholipid for solid dispersion formulations of piroxicam and was selected for further investigations.

**Table 1:** Dissolution characteristics of different solid dispersions of piroxicam (drug and phospholipid weight ratio 10:1) and piroxicam alone (mean ± SD, n ≥ 3).

Composition	Initial Dissolution Rate (% released/min)	% Released after 60 min
Control	0.4 ± 0.2	19.7 ± 4.6
Piroxicam-DMPG	6.7 ± 1.5	76.1 ± 4.1
Piroxicam-DMPC	4.9 ± 0.8	54.2 ± 11.5
Piroxicam-DPPC	4.1 ± 1.0	46.8 ± 5.1
Piroxicam-DSPC	3.1 ± 0.7	43.8 ± 4.0

### 3.2. Effect of DMPG Weight Fraction on the Dissolution Rate of Piroxicam

It is generally known that the dissolution rate of a drug may increase as a function of the carrier concentration in the solid dispersion [13,15,19]. Thus, to evaluate the effect of DMPG weight fraction on the dissolution rate of piroxicam, the solid dispersions with varying drug-to-carrier ratios (5:1, 10:1, and 15:1) were prepared and tested.

**Figure 2:**
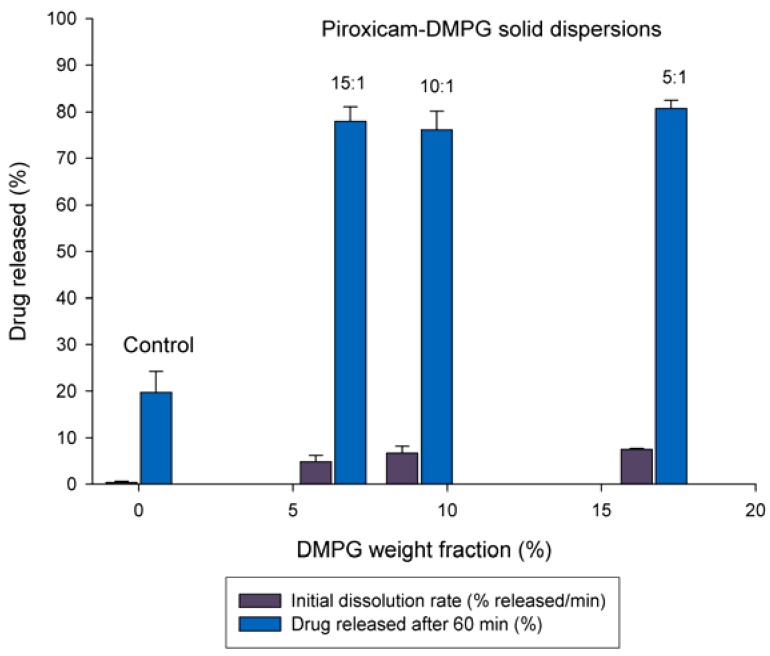
Effect of DMPG weight fraction on the dissolution parameters of piroxicam from solid dispersions at 37 ºC in distilled water.

The dissolution parameters of piroxicam as a function of the weight fraction of DMPG in these systems are plotted in Figure 2. These results revealed that although increasing the DMPG weight fraction in the solid dispersions from 6.4 to 16.7% (15:1 to 5:1) resulted in about 53% gain (from 4.9 to 7.5% released/min) in the initial dissolution rate, it had no significant (~ 5% greater) effect on the cumulative amount of piroxicam dissolved in 60 min from these systems. Such dissolution performance of DMPG-based solid dispersions is likely to be associated with a unique phase behavior of this phospholipid in diluted aqueous dispersions. Under these conditions, DMPG has been shown to undergo a sharp phase transition (instead of the chain melting process), with *T*c being increased up to about 41 ºC with decrease in the phospholipid concentration [26]. Dissolution studies were apparently performed under the analogous conditions, where DMPG exhibited an elevated *T*c similar for all the solid dispersion systems studied. As a result, the hydration of the carrier phospholipid was retarded so that no considerable difference among the formulations in terms of the cumulative amount of the drug dissolved was observed.

For comparison, the dissolution test was also performed with the 15:1 piroxicam-DMPG physical mixture. As expected, the physical mixture was found to exhibit a slightly enhanced dissolution profile when compared to that of the control. Specifically, an increase in both initial dissolution rate (about a 2-fold) and the drug limiting concentration after 60 min (by ~ 45%) was observed. This enhancing effect is commonly attributed to improved wetting properties of poorly water soluble drugs in the presence of wetting agents such as phospholipids and liposome formation in the dissolution media [5].

### 3.3. Solid-state Characterization of DMPG-based Solid Dispersions

Rearrangement of the crystal structure or complete loss of the long-range order (amorphization), or the formation of solid solutions or eutectics are among the common reasons that account for an increased dissolution rate of the APIs from solid dispersions [6]. Moreover, the model dug, piroxicam, is known to exhibit polymorphism [27,28] and is thus susceptible to various solid phase transformations during processing. Hence, to gain an insight into the mechanism of improved dissolution rate of piroxicam from DMPG solid dispersions, the solid-state properties of these systems were investigated by means of PXRD and DSC.

**Figure 3:**
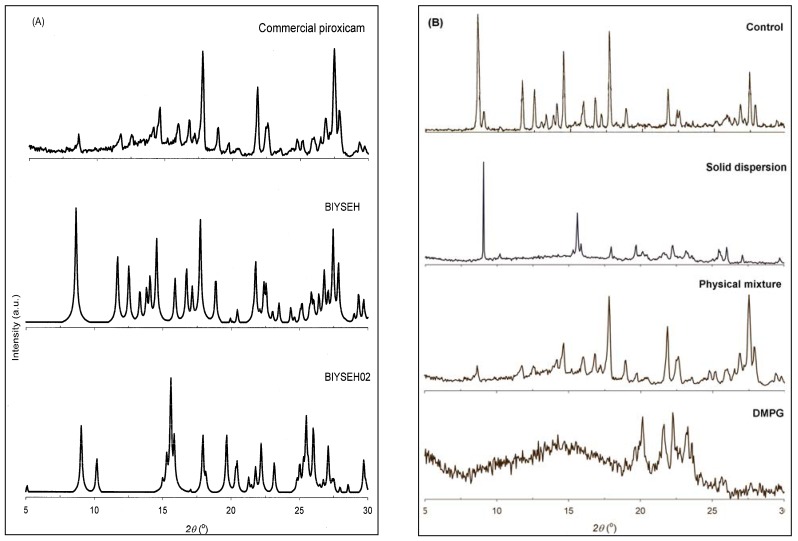
Powder X-ray diffraction patterns of (**A**) commercial piroxicam in comparison with theoretical patterns of its polymorphic forms, Form I (BIYSEH) and Form II (BIYSEH02), and (**B**) the carrier phospholipid DMPG, the 15:1 (w/w) piroxicam-DMPG physical mixture and solid dispersion *vs*. control piroxicam.

Representative powder X-ray diffraction (PXRD) patterns for the starting materials, control, physical mixture, and solid dispersions of piroxicam and DMPG are shown in Figure 3. By comparing the theoretical [29,30] and experimental PXRD patterns (Figure 3A), untreated (commercial) piroxicam was identified as Form I (BIYSEH). The PXRD pattern of the physical mixture (Figure 3B) was found to be a simple summation of those of piroxicam (Form I) and DMPG. However, the PXRD patterns yielded by the solvent-treated samples, the control and solid dispersions (Figure 3B), did not match well with the diffraction patterns of either Form I or the piroxicam-DMPG physical mixture. This result indicates that a solution-mediated phase transformation occurred during the solid dispersion formation. In particular, the control was identified as a mixture of the two polymorphic forms, Form I and Form II (BIYSEH02). This finding is consistent with the previous studies [21,27] that reported the formation of polymorphic mixtures of piroxicam during solvent-based processes. In this context, it was interesting to note that the PXRD pattern of the solid dispersion was in a reasonable agreement with that of Form II. Obviously, this metastable form of piroxicam was formed as a result of enhanced crystallization kinetics due to the presence of a solubilizing agent (DMPG) in the media. Furthermore, piroxicam (Form II) exhibited no phase transformation within the formulation upon storage at ambient conditions for three months, as verified by PXRD analysis. Form II has been shown to have a faster dissolution rate as compared to Form I [27] and thus this solid phase transformation can be linked with the enhanced dissolution kinetics of the DMPG-based formulations.

Thermal characteristics of untreated piroxicam, the control, physical mixture, and the solid dispersions are compared in Table 2. In accordance with PXRD results, untreated piroxicam showed a sharp endotherm at 201.1 ºC (onset temperature), which is consistent with the melting point of Form I [27]. Control piroxicam yielded a broader endotherm with the onset at around 199.3 ºC that corresponded to the melting point of Form II [27], further confirming the formation of a polymorphic mixture. With solid dispersions, a gradual depression in both the melting temperature and enthalpy of melting with the increase of DMPG weight fraction in the formulation was observed, signifying an increased fraction of the drug dissolved in the carrier phospholipid. It should be emphasized, however, that the 15:1 physical mixture of piroxicam (Form I) and DMPG also yielded a melting endotherm downshifted to 198.9 ºC. This implies that no conclusion regarding the polymorphic form of a drug dispersed in the carrier could be drawn solely based on the DSC analysis. Nevertheless, some correlation between the reduced melting point (and especially enthalpy of fusion) and increased initial dissolution rate of the formulations could be observed.

**Table 2:** Thermal characteristics of piroxicam and piroxicam-DMPG binary systems. Each point is the mean ± SD (n = 3).

Sample	Melting temperature (onset, ºC)	Heat of melting (∆*H*, *J/g*)
Commercial Piroxicam	201.1 ± 0.6	103.3 ± 1.0
Control Piroxicam	199.3 ± 1.1	98.5 ± 1.8
Piroxicam:DMPG physical mixture (15:1, w/w)	198.9 ± 1.6	93.7 ± 0.9
Piroxicam:DMPG solid dispersions		
15:1 (w/w)	198.4 ± 2.3	91.1 ± 1.9
10:1 (w/w)	196.8 ± 1.7	75.6 ± 2.5
5:1 (w/w)	195.2 ± 2.1	74.4 ± 3.2

### 3.4. In vivo Performance of 15:1 Piroxicam-DMPG solid Dispersion

The preliminary *in vitro* studies revealed no significant positive effect of DMPG weight fraction on the dissolution rate of piroxicam (see Section 3.2). For this reason, the 15:1 piroxicam-DMPG solid dispersions (*i.e.*, the formulations with the highest drug loading) were utilized for *in vivo* studies.

**Figure 4:**
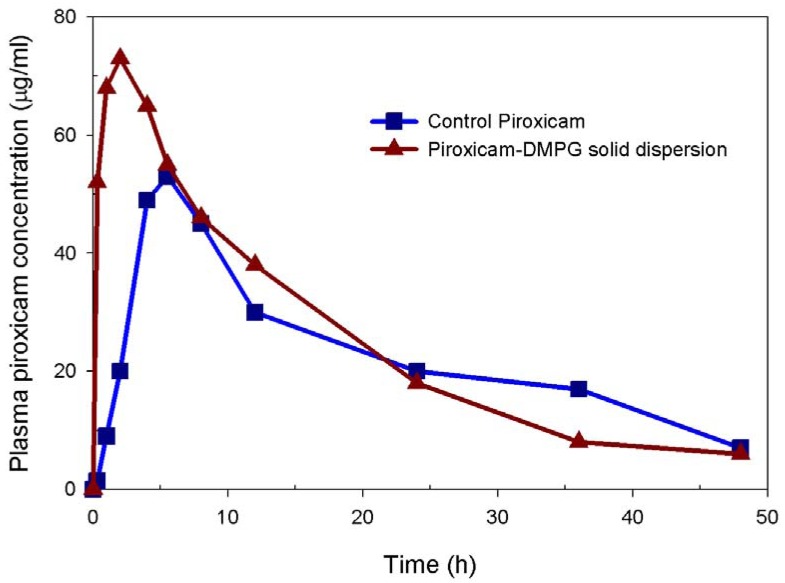
Representative plasma concentration-time profiles for two piroxicam formulations. The DMPG-based solid dispersion formulations of piroxicam were administered orally to rats and compared to the same dose of control piroxicam (20 mg/kg).

The representative plasma piroxicam concentration *vs.* time profiles of the two oral formulations, the control and the 15:1 solid dispersion, after the single dose are plotted in Figure 4; the main pharmacokinetic parameters for these formulations are summarized in Table 3. The analysis of the data shows that the piroxicam-DMPG solid dispersion exhibits a superior pharmacokinetic profile, reaching the peak plasma concentration (*c*_max_) of about 53 μg/mL rather fast (*T*_max_ = 2 h). The concentration maximum of the control was about 39 μg/mL and 1.4-fold lower than that of the solid dispersion formulation and was reached within a much longer period of time (approximately 5.5 h), indicating the differences in absorption properties (and thus bioavailability) of these piroxicam formulations. The other pharmacokinetic parameters, including AUC_0-48_, elimination half life and mean residence time (MRT), were comparable for both formulations.

Therefore, referring to the dissolution behavior of these model systems, as described above, a correlation between *in vitro* dissolution and *in vivo* bioavailability of piroxicam can be found. In particular, the increased rate and extent of *in vitro* dissolution appeared to correlate with the enhanced bioavailability parameters such as *c*_max_ and *T*_max_. The enhanced human absorption of NSAIDs is usually associated with an improved analgesic effect [31]. In this context, piroxicam-DMPG solid dispersions are expected to exhibit a superior pharmacological effect.

**Table 3:** Pharmacokinetic parameters (mean ± SD, n = 3) of two piroxicam formulations following single-dose oral administration in rats.

Parameters	Formulation	
	Control	DMPG-based solid dispersion (15:1, w/w)
Peak plasma concentration (*c*_max_, μg/mL)	38.9 ±14.3	53.3 ± 15.1
Time to peak concentration (*T*_max_, h)	5.5 ± 2.1	2.0 ± 0.0*
AUC_0-48 _ (μg/mL . h)	921 ± 207	1210 ± 254
Elimination half life (T_1/2_, h)	13.0 ± 2.7	14.5 ± 5.6
Mean residence time (MRT, h)	25.9 ± 5.5	27.7 ± 8.2

* Significantly different from control piroxicam (p < 0.05).

## 4. Conclusions

Four phospholipids, dimyristoylphosphatidylglycerol (DMPG), dimyristoylphosphatidylcholine (DMPC), dipalmitoylphosphatidylcholine (DPPC), and distearoylphosphatidylcholine (DSPC), were assessed as potential carriers/additives for rapidly dissolving solid formulations of piroxicam (BCS class II drug). Among these phospholipids, DMPG was shown to be the most potent dissolution rate enhancer for the model drug. However, due to a peculiar thermal behavior of DMPG in diluted aqueous dispersions, an extended study on the phospholipids concentration/dissolution rate relationships is recommended in order to rationally design a formulation. Furthermore, a correlation between the rate and extent of *in vitro* dissolution and the bioavailability parameters (*c*_max_ and *T*_max_) of piroxicam was demonstrated. Hence, DMPG-based formulations proved to be the most promising delivery systems for oral bioavailability enhancement of BCS Class II drugs such as piroxicam.
